# Analyzing editosome function in high-throughput

**DOI:** 10.1093/nar/gkaa658

**Published:** 2020-08-05

**Authors:** Cristian Del Campo, Wolf-Matthias Leeder, Paul Reißig, H Ulrich Göringer

**Affiliations:** Molecular Genetics, Technical University Darmstadt, Schnittspahnstr. 10, 64287 Darmstadt, Germany; Molecular Genetics, Technical University Darmstadt, Schnittspahnstr. 10, 64287 Darmstadt, Germany; Molecular Genetics, Technical University Darmstadt, Schnittspahnstr. 10, 64287 Darmstadt, Germany; Molecular Genetics, Technical University Darmstadt, Schnittspahnstr. 10, 64287 Darmstadt, Germany

## Abstract

Mitochondrial gene expression in African trypanosomes and other trypanosomatid pathogens requires a U-nucleotide specific insertion/deletion-type RNA-editing reaction. The process is catalyzed by a macromolecular protein complex known as the editosome. Editosomes are restricted to the trypanosomatid clade and since editing is essential for the parasites, the protein complex represents a near perfect target for drug intervention strategies. Here, we report the development of an improved *in vitro* assay to monitor editosome function. The test system utilizes fluorophore-labeled substrate RNAs to analyze the processing reaction by automated, high-throughput capillary electrophoresis (CE) in combination with a laser-induced fluorescence (LIF) readout. We optimized the assay for high-throughput screening (HTS)-experiments and devised a multiplex fluorophore-labeling regime to scrutinize the U-insertion/U-deletion reaction simultaneously. The assay is robust, it requires only nanogram amounts of materials and it meets all performance criteria for HTS-methods. As such the test system should be helpful in the search for trypanosome-specific pharmaceuticals.

## INTRODUCTION

Human African trypanosomiasis (HAT), also known as sleeping sickness, is a neglected tropical disease. Despite a decreasing number of new infections in recent years, still 70 million people in 36 African countries are at risk of becoming infected (1). In response to the declining incidence, the World Health Organization (WHO) has targeted HAT for elimination as a public health problem by 2020 (2). However, a similar situation was already achieved in the 1960s, which was followed by a reduction in surveillance and control activities and as a consequence the disease resurfaced again in the 1990s (3). This advocates that efforts to develop trypanosome-specific diagnostics and therapeutics should not be suspended (4). Five drugs are currently used in the treatment of HAT (5). All of them are toxic to different degrees (1). In addition, they suffer from a multitude of complications including parenteral administration (6), poor efficacy, clinical side effects and increasing levels of resistance (7–9). As a consequence, a novel and improved HAT-therapeutic would be of great value (10). Causative agent of HAT is *Trypanosoma brucei*, an extracellular, single-cell parasite. The organism proliferates in the blood and lymph fluid and is capable of crossing the blood-brain barrier where it induces a wide spectrum of neurological symptoms (5). Left untreated, progressive neurological deterioration leads to coma and death (11).

Mitochondrial gene expression in trypanosomes and related pathogens requires an RNA-editing reaction in which sequence-deficient, non-translatable primary transcripts are converted into translatable mRNAs by the site-specific insertion and deletion of exclusively U-nucleotides (12, reviewed in 13). The reaction pathway is catalyzed by a unique, high molecular mass multienzyme complex known as the editosome (14,15, reviewed in 16). Editosomes execute the processing reaction in a cascade of enzyme-mediated steps, which include RNA-chaperone, endo- and exonuclease, terminal uridylyl transferase (TUTase) and RNA-ligase activities. Furthermore, the process is mediated by small, non-coding RNAs, termed guide (g)RNAs. They act as templates and direct the U-insertion/deletion by anti-parallel base pairing (17,18). Several features make the editosome a prime drug target. First, RNA-editing is an essential pathway in trypanosomes. Second, the editosome is uniquely present in the parasite, with no corresponding enzyme complex in the host. Third, the reaction cycle involves multiple enzyme reactions, all representing potential drug-interference points, and fourth, the high molecular mass protein complex (0.8MDa) offers a large drug-binding landscape (15). Despite these favorable features, only very few RNA-editing inhibitors have been identified (15, 19–23). Moreover, only three studies have systematically searched for RNA-editing inhibiting compounds, which primarily is due to the lack of a robust, high-throughput compatible assay system. Available formats include an electrochemiluminescent RNA aptamer-based test (24), a hammerhead ribozyme (HHR)-driven reporter assay connected to Förster resonance energy transfer (FRET)-detection (25) and a second FRET-based system to detect RNA-ligase 1-specific inhibitors (23). Despite the fact that the described assays successfully identified RNA editing-specific inhibitors, the different formats also pose limitations. These range from relying on indirect measuring principles to focusing exclusively on one step of the reaction cycle. Most importantly however, none of the methods is able to provide a complete and quantitative analysis of all editosome-mediated catalytic conversion steps.

**Figure 1.**
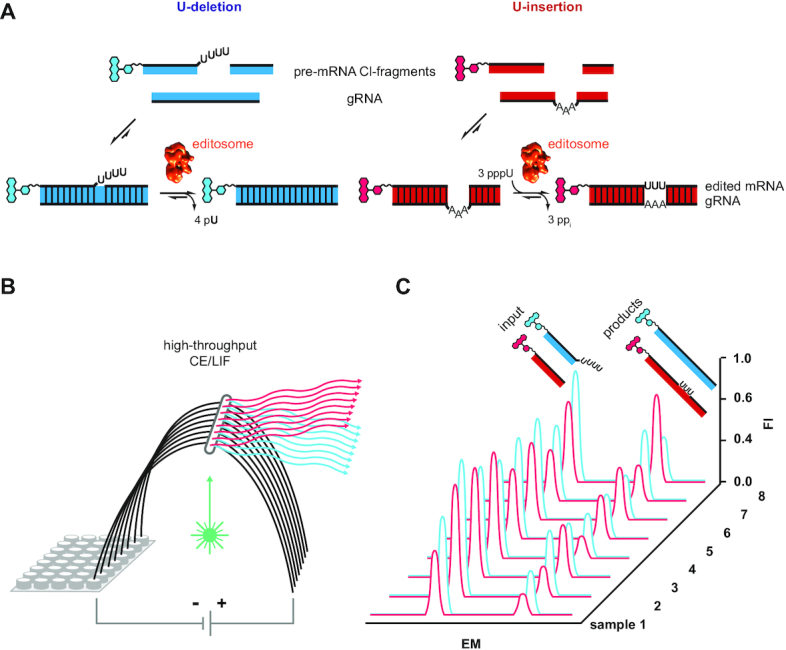
Work flow of the fluorescence-based insertion/deletion editing (FIDE)-assay. (**A**) The test system uses short, synthetic oligoribonucleotides mimicing the pre-edited mRNAs and gRNAs of the editing reaction. Two sets of RNA-oligonucleotides are synthesized to monitor the U-insertion and U-deletion reaction separately. Pre-mRNA molecules are introduced as pre-cleaved RNAs (5′-cleavage (Cl)-fragment, 3′-Cl-fragment) to sidestep the rate-limiting endonucleolytic cleavage reaction. Fluorophore-derivatized versions (chemical ring systems in red and blue) of the pre-mRNA oligonucleotides are annealed to their base-complementary gRNAs to form trimolecular pre-mRNA/gRNA hybrid RNAs, which represent the substrates of the editing reaction. Upon incubation with editosomes (cryo-EM structure of the editosome (15) in red) the pre-mRNAs are edited by the insertion of three U-nt or the deletion of four Us. (**B**) Reactions are performed in multiwell plates and are subsequently analyzed by automated capillary electrophoresis (CE) coupled to laser-induced fluorescence (LIF) detection. Using different fluorophores for the U-insertion and U-deletion, both reactions can be monitored simultaneously. (**C**) The resulting electropherograms are peak integrated, facilitating a quantitative side-by-side comparison of every product and intermediate of the two reactions. The usage of multi-capillary CE-instruments provides a highly parallelized, high-throughput environment, in which hundreds of samples can be analyzed at the same time. As such the assay is well-suited for drug-screening purposes. EM: electrophoretic migration time; FI: fluorescence intensity.

Here, we present a new, high-throughput RNA-editing screening assay. The test system is a modified version of the established *in vitro* RNA-editing assays (26–30), but instead of using radioactively labeled substrate-RNAs it utilizes fluorophore-derivatized RNA-substrates (Figure 1). This enables the usage of automated, highly parallelized capillary electrophoresis (CE) instruments coupled to laser-induced fluorescence (LIF) detection systems. We optimized the work flow of the assay by adjusting the material quantities and reaction volume to multiwell-plate formats, by increasing the RNase-stability of the substrate-RNAs and by devising a multiplex fluorophore-labeling regime to scrutinize the U-insertion and U-deletion reaction in one reaction vial using chemically identical U-insertion/U-deletion substrate RNAs. The assay is robust, it satisfies all signal-to-noise criteria for high-throughput screening methods and it is able to derive quantitative data for every reaction step of the catalytic conversion. To validate the assay, we surveyed UTP-analogs for their aptitude to inhibit the U-insertion reaction.

## MATERIALS AND METHODS

### Chemical RNA-synthesis and postsynthetic processing

RNA-oligonucleotides were synthesized by solid-phase synthesis on controlled pore glass (CPG)-beads (50nmol synthesis scale) using 2′*-O*-(*tert*-butyl)dimethylsilyl (TBDMS)-protected phosphoramidites. 5-Carboxytetramethyl-rhodamine (TAMRA, λ_ex_ 546 nm, λ_em_ 579 nm), 6-hexachloro-fluorescein (HEX, λ_ex_ 535 nm, λ_em_ 556 nm) and 6-carboxy-fluorescein (FAM, λ_ex_ 492 nm, λ_em_ 517 nm) were chosen as fluorophore substituents and were introduced post-synthetically either at the 5′- or 3′-ends of the different oligoribonucleotides. For that the RNA-oligos were synthesized to contain either a 5′- or 3′-terminal C6-aminolinker. The primary amino group was then conjugated to carboxyl functional groups of FAM, TAMRA and HEX using an EDC (1-ethyl-3-(3-dimethylaminopropyl)carbodiimide)-mediated coupling reaction. As additional modifications, some RNAs were synthesized to include up to six phosphorothioate (PS) backbone modifications to increase their RNase-stability. Similarly, some of the 3′-pre-mRNA oligonucleotides were 3′-end modified by introducing a hexamethylene-amino-linker. Base-protecting groups were removed at mild conditions using NH_4_OH/EtOH (3:1) at RT and 2′-silyl protecting groups were removed using neat triethylamine trihydrofluoride. All RNA-oligonucleotides were HPLC-purified, analyzed by mass-spectrometry and further scrutinized in denaturing polyacrylamide gels (Supplementary Figure S1). RNA-concentration were derived from UV-absorbency measurements at 260 nm (*A*_260_) using the molar extinction coefficients (ϵ in l/mol cm) listed below. Oligoribonucleotides representing 3′-pre-mRNA fragments were enzymatically 5′-phosphorylated using T4-polynucleotide kinase (T4-PNK) and ATP using standard conditions. The following RNA-oligonucleotides were synthesized:

**Table:** 

U-insertion editing:	
5′-Cl18	GGAAGUAUGAGACGUAGG (197300)
5′-TAMRA_Cl18	TAMRA-(CH_2_)_6_-GGAAGUAUGAGACGUAGG (247200)
3′-Cl13	AUUGGAGUUAUAG-(CH_2_)_6_-NH_2_ (144200)
3′-Cl13_FAM	AUUGGAGUUAUAG-(CH_2_)_6_-FAM (173500)
gRNA_ins_	CUAUAACUCCGAUAAACCUACGUCUCAUACUUCC (328200)
U-deletion editing:	
5′-Cl22	GGAAAGGGAAAGUUGUGAUUUU (237100)
5′-FAM_Cl22	FAM-(CH_2_)_6_-GGAAAGGGAAAGUUGUGAUUUU (284900)
3′-Cl15	GCGAGUUAUAGAAUA-(CH_2_)_6_-NH_2_ (167300)
3′-Cl15_TAMRA	GCGAGUUAUAGAAUA-(CH_2_)_6_-TAMRA (209600)
gRNA_del_	GGUUCUAUAACUCGCUCACAACUUUCCCUUUCC (305200)
HEX_mRNA_del_	HEX-(CH_2_)_6_-GGAAAGGGAAAGUUGUGAUUUUGCGAGUUAUAGAAUA (480800)
U-insertion editing/non-natural RNAs:	
5′-FAM_synCl14	FAM-(CH_2_)_6_-AAAGGAAAUAUAGU (20500)
3′-synCl16	AGGUGAUUCCAUUGAG (181100)
syngRNA_ins_	CUCAAUGGAAUCACCUAAAACUAUAUUUCCUUU (363200)
U-insertion editing/non-natural RNAs/PS-modified (*):	
5′-FAM_modCl14	FAM-(CH_2_)_6_-AAAGGAAAU*A*U*A*GU (20500)
3′-modCl16	AG*G*U*GAUUCCAUUGAG-(CH_2_)_6_-NH_2_ (181100)
modgRNA_ins_	C*U*C*AAUGGAAUCACCUAAAACUAUAUUUCC*U*U*U (363200)
U-deletion editing/non-natural RNAs:	
5′-FAM_synCl17	FAM-(CH_2_)_6_-AAAGGAAAUAUAGUUUU (232600)
3′-synCl16	AGGUGAUUCCAUUGAG (181100)
syngRNA_del_	CUCAAUGGAAUCACCUAAAACUAUAUUUCCUUU (363200)
U-deletion editing/non-natural RNAs/PS-modified (*):	
5′-FAM_modCl17	FAM-(CH_2_)_6_-AAAGGAAAU*A*U*A*GUUUU (232600)
3′-modCl16	AG*G*U*GAUUCCAUUGAG-(CH_2_)_6_-NH_2_ (181100)
modgRNA_del_	C*U*C*AAUGGAAUCACCUACUAUAUUUCC*U*U*U (317000)
RNA-size standards:	
FAM_High44	FAM-(CH_2_)_6_-CUAGUACUCUCAUCAACAUAAGUCUCAUACUUCCGACAUGCACG (493200)
HEX_Low11	HEX-(CH_2_)_6_-ACUUCAACUCG (147000)

### Formation of pre-mRNA/gRNA-hybrid RNAs

Trimolecular pre-mRNA/gRNA-hybrid molecules were generated by hybridization of the individual 5′- and 3′-pre-mRNA oligoribonucleotides to base complementary gRNA molecules. For that, equimolar amounts (4 pmol) of the three RNA-oligonucleotides were combined in a final volume of 0.1 ml 10 mM Tris/HCl, 1mM Na_2_EDTA (TE) pH 7.5 followed by denaturation at 75°C for 2 min. Denatured oligoribonucleotides were annealed by cooling samples to 27°C at a rate of 0.08°C/s. Formation of the pre-mRNA/gRNA-hybrid RNAs was verified by electrophoresis in native 15% (w/v) polyacrylamide gels followed by staining with Toluidine blue O. Stained gels were densitometrically analyzed.

### Editosome purification

Editosomes were purified from insect-stage African trypanosomes (*Trypanosoma brucei*), which were grown at 27°C in SDM-79 medium (31) in the presence of 10% (v/v) fetal calf serum. The complexes were either purified by tandem-affinity purification (TAP) (15,32,33) using transgenic *T. brucei* cell lines that conditionally express TAP-tagged versions of editosomal proteins or from mitochondrial detergent extracts of wildtype trypanosomes (strain Lister 427) as described in (34). The following transgenic parasite strains were used: *T. brucei* 29–13 KREPA4/TAP (35), *T. brucei* 29–13 KREPA3/TAP (15) and *T. brucei* 29–13 KRET2/TAP (36,37). Typically about 6 × 10^11^ parasites were disrupted at isotonic, near-native conditions and cell lysates were processed using IgG- and calmodulin-affinity chromatography resins. Protein concentrations were determined by Bradford dye binding and the protein inventory of the different isolates was analyzed by tandem mass spectrometry (nanoLC–MS/MS).

### FIDE—fluorescence-based insertion/deletion editing

RNA-editing reactions were assembled in a 30μl volume in editing buffer (EB: 20 mM HEPES pH 7.5, 10 mM MgCl_2_, 30 mM KCl) supplemented with 0.5 mM DTT and 0.5 mM ATP. For the U-insertion assay, additional 0.1 mM UTP or analogs of UTP were added. Reactions contained 0.4 pmol pre-annealed pre-mRNA/gRNA hybrid RNA and 0.2–0.4 pmol of editosomes. Samples were incubated at 27°C for 3 h after which 15 fmol of two size-standard oligoribonucleotides (FAM_High44 and HEX_Low11) were added. Reactions were stopped by phenol/chloroform extraction and the processed RNAs were EtOH precipitated. Samples were immediately centrifuged for 30 min at 13 000 rpm (4°C) and RNA pellets were washed (70% (v/v) EtOH), dried *in vacuo* and resolved in 100 μl of Hi-Di™ formamide. Samples were heat denatured at 95°C for 2 min, snap-cooled and separated by capillary electrophoresis (CE) at 12 kV for 25 min using a POP-6 polymer (Thermo Scientific). ‘One-pot’ U-insertion/U-deletion reactions were assembled by annealing of the U-insertion and U-deletion pre-mRNA/gRNA-hybrid RNAs in separate reaction vials. Equimolar amounts (0.2 pmol) of each hybrid RNA were then combined with 0.2–0.4 pmol of editosomes in EB in the presence of 0.5 mM ATP, 0.1 mM UTP and 0.5 mM DTT in a final volume of 30 μl. All incubation and processing steps of the one-pot samples were performed as above. Down-scaled U-insertion and U-deletion assays were performed with 10 fmol of pre-mRNA/gRNA hybrid RNAs and 2.5 fmol of editosomes in a volume of 4 μl. The reduced amounts of material made it possible to skip the phenol extraction and EtOH-precipitation steps of the work flow and enabled a direct sample application onto the CE-system. We also confirmed that the FIDE-assay can be performed in a non-precleaved fashion. For that we used the pre-mRNA mimicking oligoribonucleotide HEX_mRNA_del_ in conjunction with gRNA_del_.

### Data processing

Raw data from the CE-runs (relative fluorescence units (RFU) and electrophoretic migration times in seconds) were converted into tab-delimited text files and imported either into OriginPro v8.5 (OriginLab Corporation) or MultiGauge v3.0 (Fuji Photo Film, Co.). The quality of the CE-separations was assessed by evaluating the signal-to-noise (S/N) ratio as S/N = (μ_signal_/μ_noise_)/∂_noise_ (μ = mean; ∂ = standard deviation). The data were baseline corrected and individual peaks were assigned by comparison to the unedited peak of a mock reaction using the electrophoretic migration times of two 11nt- and 44nt-long calibration oligoribonucleotides (FAM_High44 and HEX_Low11) as reference points. Peaks were integrated using a Riemann sum approximation to derive RNA-editing activity values (EA) defined as the ratio of the peak area (*A*) of the fully edited product (*A*_FE_) versus the sum of all peaks, i.e. the unedited (*A*_UE_), partially edited (*A*_PE_) and fully edited (*A*_FE_): EA = *A*_FE_/∑(*A*_UE_, *A*_PE_, *A*_FE_). EA-values divided by the mass of editosomes represent the specific RNA-editing activity of the editosome preparation in EA/ng. By computing the ratio between the area of each peak and the total integrated area, the fraction of each reaction intermediate was calculated. In the case of 'one-pot' RNA-editing assays, raw data from the electropherograms were first imported into ShapeFinder (38) for baseline correction and matrixing in order to correct for the unique contribution of each fluorophore to the signal intensity in each fluorescence channel. The quality of the HTS-experiment was assessed by calculating the mean screening window coefficient (*Z*') as *Z*' = 1 – (3∂_control+_ + 3∂_control–_)/|μ_control+_-μ_control-_| (μ = mean, ∂ = standard deviation, control+ = positive control, control– = negative control) (39).

### UV-hyperchromicity measurements

Absorbance versus temperature profiles (melting curves) of the different pre-mRNA/gRNA hybrid RNAs were recorded at 260 nm (*A*_260_) using a thermoelectrically controlled UV-spectrophotometer. The temperature was scanned at a heating rate of 1°C/min at temperatures between 20 and 90°C. Absorbance values were recorded with an average time of 0.5 s and data were collected every 0.1°C. Samples contained 1 μM pre-mRNA/gRNA hybrid RNA in 10 mM Na-cacodylate pH 6.8, 65 mM NaCl. Half-maximal melting temperatures (*T*_m_) were calculated from first-derivative plots of absorbance versus temperature δ*A*_260_/δ*T* = f(*T*) (21).

### Ribonucleolytic degradation

The RNase-sensitivity of unmodified and phosphorothioate-modified 5′-Cl oligoribonucleotides (U-deletion: 5′-FAM_Cl22, 5′-FAM_synCl17, 5′-FAM_modCl17; U-insertion: 5′-TAMRA-Cl18, 5′-FAM_synCl14, 5′-FAM_modCl14) was tested by incubation of 0.4pmol RNA-oligonucleotide with 0.2 pmol of editosomes in 30 μl EB for 3 h at 27°C. Samples were processed and analyzed as described above.

## RESULTS AND DISCUSSION

### Starting analysis—characterization of fluorophore-labeled pre-edited mRNA/gRNA hybrid RNAs

**Figure 2.**
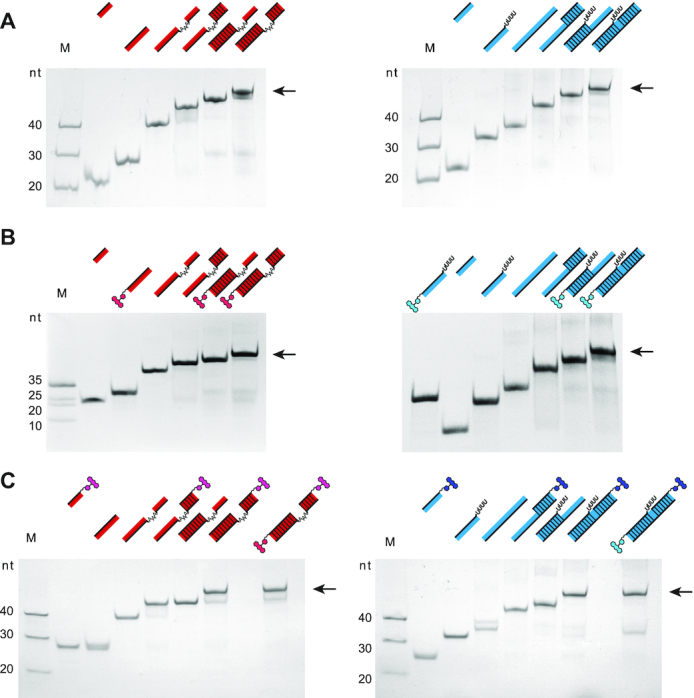
Pre-mRNA/gRNA-hybrid formation. Gel-electrophoretic analysis of the formation of trimolecular pre-mRNA/gRNA hybrid RNAs comparing non-modified RNA-oligonucleotides (**A**) with single 5′- or 3′-fluorophore-labeled RNAs (**B, C**) and dual-modified (5′ and 3′) RNAs (C). U-insertion RNAs are in red. U-deletion RNAs are in blue. All gel-electrophoretic separations show the 3′-pre-mRNA cleavage fragment (3′-Cl), the 5′-pre-mRNA cleavage fragment (3′-Cl) and the corresponding gRNA-oligoribonucleotide next to the two bimolecular complexes (5′-Cl/gRNA; 3′-Cl/gRNA) and the final trimolecular (5′-Cl/3′-Cl/gRNA) annealing product (arrow). Fluorophore-subtituents are shown as chemical ring systems in red and blue. M = marker, nt = nucleotides.

**Figure 3.**
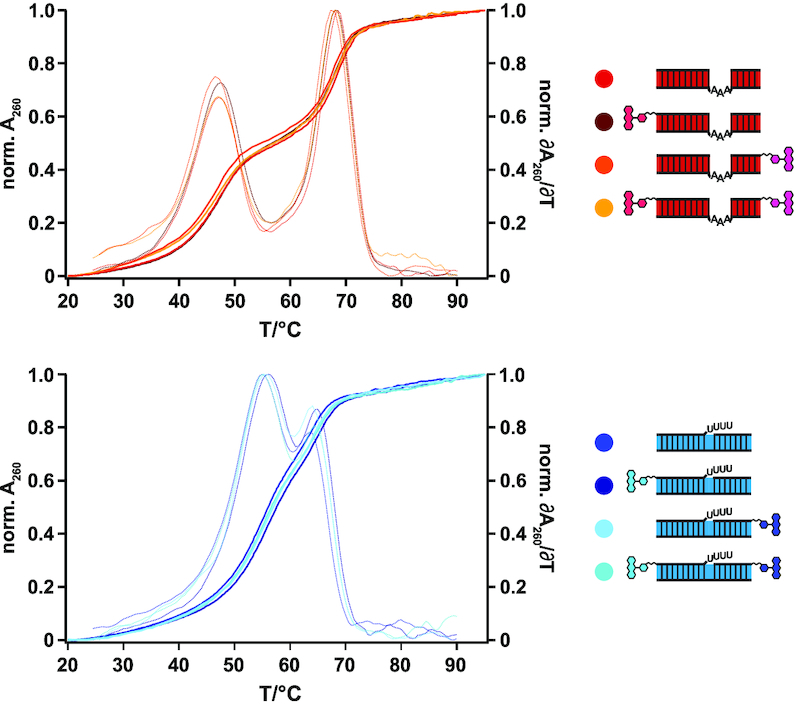
Thermodynamic stabilities of pre-mRNA/gRNA-hybrid RNAs. Comparison of the UV-melting (*A*_260_ = f(*T*)) and first-derivative profiles (δ*A*_260_/δ*T* = f(*T*)) of non-labeled and fluorophore-labeled pre-mRNA/gRNA hybrid RNAs. Red/yellow: U-insertion pre-mRNA/gRNA hybrid molecules. Dark/light blue: U-deletion pre-mRNA/gRNA hybrid RNAs. Fluorophore-positions are depicted as chemical ring systems in red and blue.

The established U-insertion/U-deletion *in vitro* RNA-editing assay (26–30) represents a chemically simplified experimental system in which both, the substrate pre-edited mRNAs as well as the *trans*-acting gRNAs are mimicked by short oligoribonucleotides (13–22nt) (Figure 1). Furthermore, only single editing sites are embedded in each of the two pre-mRNAs, which in the case of the U-insertion assay is edited in a gRNA-dependent manner by incorporating three U-nucleotides (nt). During the U-deletion reaction four U-nt are removed. Furthermore, in order to sidestep the rate-limiting endonucleolytic cleavage of the pre-mRNA molecules, the corresponding oligoribonucleotides are “pre-cleaved". This generates 5′- and 3′-pre-mRNA cleavage fragments, which are abbreviated as 5′-Cl and 3′-Cl (Figure 1). The 5′-Cl oligoribonucleotides are typically 5′-(^32^P)-labeled to enable a radioactive readout of the assay. To convert the system into a fluorescence-based assay we covalently attached different fluorogenic side groups to either the 5′- or 3′-terminal phosphate groups of the two Cl-oligoribonucleotides. 5-Carboxytetramethylrhodamine (5-TAMRA, 430g/mol, λ_ex_ 546 nm, λ_em_ 579 nm), 6-carboxy-fluorescin (FAM, 750 g/mol, λ_ex_ 492 nm, λ_em_ 517 nm) and 6-hexachlorofluorescein (HEX, 680g/mol, λ_ex_ 535 nm, λ_em_ 556 nm) were chosen as fluorophores. The coupling was performed post-synthetically using a 6-carbon atom spacer to minimize sterical clashes between the fluorophore moieties and the RNA-backbone. Since RNA-modifications can alter the physico-chemical properties of polynucleotides especially in the case of highly hydrophobic side-groups, we analyzed whether the different fluorophores affect the formation of the pre-mRNA/gRNA–hybrid RNAs. Antiparallel base-pairing between the pre-mRNA Cl-fragments and the corresponding gRNAs represents the first step in both *in vitro* editing reactions and the formation of the bi- and trimolecular RNA-hybrid molecules can be scrutinized by native polyacrylamide gel-electrophoresis. As shown in Figure 2A, all non-modified oligoribonucleotides anneal rapidly, i.e. within 10 min and to completion (≥97%). No difference between the U-insertion and U-deletion RNA-hybrids is detected. The same result is obtained for all fluorophore-modified oligoribonucleotide complexes (Figure 2B/C). Independent of the type of fluorophore (TAMRA, FAM, HEX) and independent of the covalent attachment site (5′ or 3′) all possible bi- and trimolecular pre-mRNA/gRNA complexes are formed with yields ≥95%. This verifies that both, the kinetic behaviour and the hydrogen bonding capacity of the different oligoribonucleotides are not affected by any of the fluorophore substituents. To broaden the analysis we further interrogated the thermodynamic stability of the fluorophore-modified hybrid RNAs. For that we recorded UV-melting profiles between 20 and 90°C. Figure 3 shows a representative result. As demonstrated before (21), non-modified versions of the insertion and deletion pre-mRNA/gRNA hybrids melt with two well separated transitions with *T*_m_-values of 68 ± 1.5°C and 47 ± 1.5°C for the U-insertion complex and 64 ± 1.5°C and 55 ± 1.5°C for the U-deletion hybrid. Importantly, all fluorophore-substituted versions of the two trimolecular RNA-complexes show qualitatively and quantitatively identical profiles. The derived *T*_m_-values deviate ≤1°C from the non-fluorophore substituted counterparts and as before, this behaviour is affected neither by the chemical nature nor by the structural positioning of the fluorophore moieties. For a complete list of all measured *T*_m_-values see Supplementary Table S1.

### FIDE—fluorescence-based insertion/deletion RNA-editing

**Figure 4.**
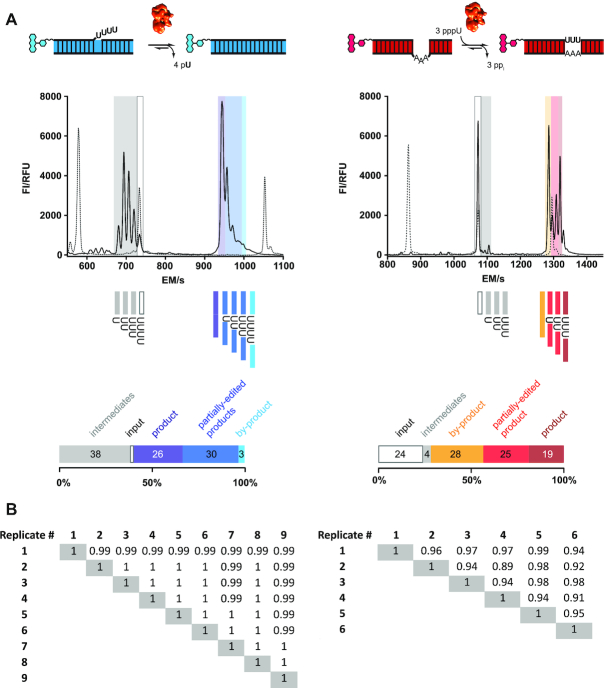
Fluorescence-based insertion/deletion RNA-editing (FIDE). (**A**) Left: capillary electrophoretic (CE)-separation of the products of a U-deletion *in vitro* RNA-editing reaction using a 5′-FAM fluorophore-modified pre-mRNA cleavage fragment (5′-FAM_Cl22). The different shadings in blue and grey highlight the input 5′-FAM_Cl22-fragment, reaction intermediates, partially edited RNAs, by-products and the fully edited -4U reaction product. Right: CE-trace of a U-insertion editing reaction using a 5′-TAMRA-fluorophore-modified pre-mRNA cleavage fragment (5′-TAMRA_Cl18). Shadings in gray, yellow and red mark the input 5′-TAMRA_Cl18 pre-mRNA fragment, reaction intermediates, partially edited RNAs, by-products and the fully edited +3U insertion product (a reference chart of all expected RNA-molecules is shown in Supplementary Figure S2). Peak area integration allows the calculation of percental quantities for every RNA-species as shown in the bar-plots below the electropherograms. FI = fluorescence intensity, RFU = relative fluorescence units; EM = electrophoretic migration time in sec. Dashed lines/peaks (from left to right) represent the electrophoretic elution positions of the 11nt marker-oligoribonucleotide HEX_Low11, the unprocessed 5′-Cl oligoribonucleotides and the 44nt marker-oligoribonucleotide FAM_High44. (**B**) Left: Pearson correlation coefficients (*p*) derived from the pairwise comparison of 9 technical replicates (#1 to #9) of a FIDE U-insertion assay. Right: Pearson correlation coefficients derived from the pairwise comparison of six biological replicates (#1 to #6) of a FIDE U-deletion assay. Each of the CE-traces consists of 1750 data points, which were compared to each other.

Next, we analyzed whether the fluorescently labeled pre-mRNA/gRNA hybrid RNAs are recognized by editosomes to act as *in vitro* RNA-editing substrates. For that we incubated fluorophore-modified insertion- and deletion-type pre-mRNA/gRNA hybrid RNAs with purified editosomes. Since the editing reaction is catalyzed in multiple enzyme-driven steps, we analyzed both, multiple turnover (editosomes<RNA-substrate) as well as single turnover conditions (editosomes ≥ RNA-substrate). This enabled us to identify the products of the catalytic conversion as well as all intermediates and side-products. After the reaction, samples were analyzed by automated capillary electrophoresis (CE) coupled to a laser-induced fluorescence (LIF) readout. Figure 4A shows representative electropherograms in which the recorded fluorescence intensity (FI) is plotted as a function of the electrophoretic migration time (EM). The U-deletion experiment was conducted with a FAM-derivatized pre-mRNA/gRNA hybrid and the electropherogram shows all 10 expected peaks: the 5′-Cl input RNA (+4U), all U-deletion intermediates of the 5′-Cl fragment as well as the fully edited and ligated -4U product. Additional RNA-species include the unedited 5′Cl/3′Cl-ligation product and all partially edited mRNAs in which only one, two or three Us have been deleted. In a similar fashion, the U-insertion assay was performed with a TAMRA-substituted pre-mRNA/gRNA hybrid. Figure 4A shows all resolved RNA-species including the reaction input, the +3U fully-edited product and the +1U and +2U partially edited RNAs. A reference chart of all expected RNA-species is shown in Supplementary Figure S2. Since the individual RNAs are resolved with nucleotide resolution, a relative quantification of every RNA-species was derived by calculating the area under each peak (peak integration), which enabled the calculation of percental RNA-editing activities (Figure 4A) or of specific RNA-editing activities per mass of editosomes. An absolute (molar) quantification was accomplished by spiking the samples with known quantities (15 fmol) of two 11nt and 44nt long calibration oligoribonucleotides (HEX_Low11, FAM_High44). Ultimately the data can be used to derive RNA-editing activity units (EU) defined as the catalytic conversion of 1pmol pre-mRNA/gRNA–hybrid RNA into fully edited mRNA in 1 h at 27°C. To test the reproducibility of the fluorescence-based assay, we compared up to nine technical and six biological replicates including editosome preparations from different *T. brucei* genetic backgrounds. The data were analyzed in a bivariate correlation analysis to calculate Pearson's correlation coefficients (*p*). Figure 4B shows a summary of the results. Technical replicates are characterized by *p*-values ≥0.99 and biological replicates by *p*-values ≥0.89 demonstrating a high level of experimental robustness. A comparison of the fluorophore CE/LIF-based *in vitro* assays with the standard radioactive, slab gel-based assays resulted in *p*-values ≥0.86 (Supplementary Figure S3).

### Optimization I—multiplexing and downscaling

**Figure 5.**
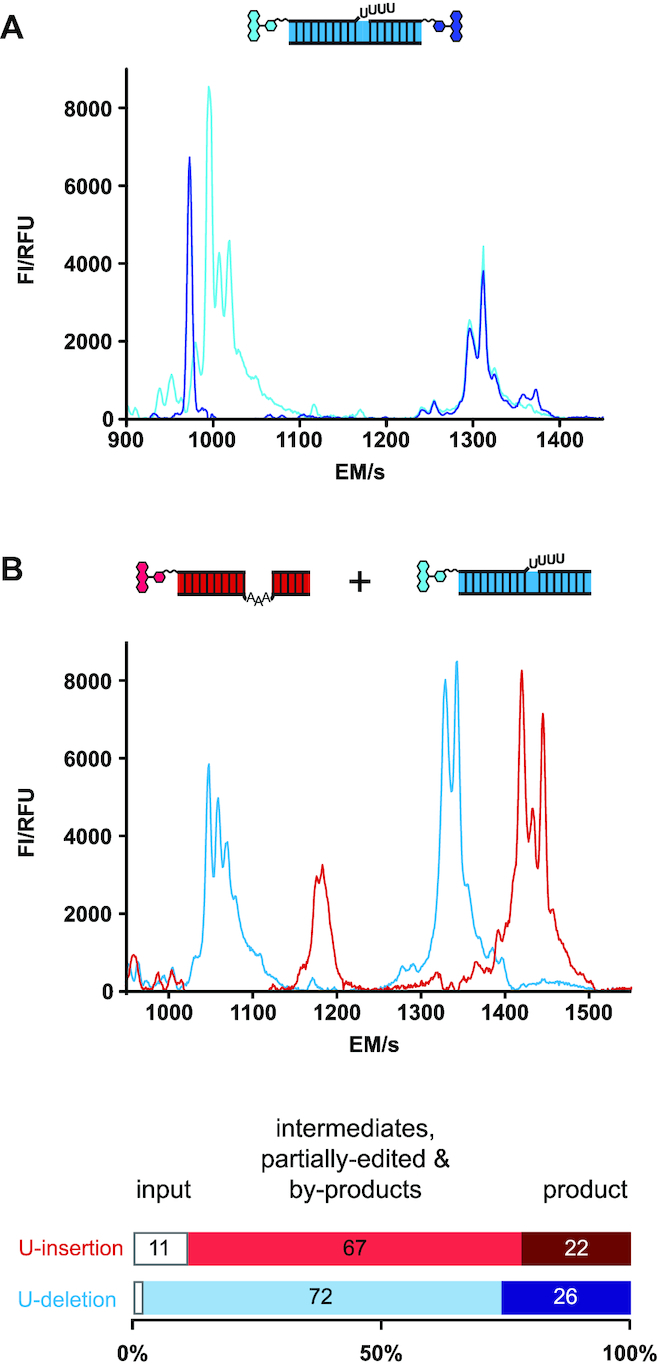
FIDE-multiplexing. (**A**) Cartoon of a dual fluorophore-labeled U-deletion RNA-editing substrate RNA, assembled by using a 5′-FAM-labeled 5′-pre-mRNA Cl-fragment (5′-FAM_Cl22) and a 3′-TAMRA-modified 3′ Cl-fragment (3′-Cl15_TAMRA). The electropherogram shows the FAM-derived CE/LIF-trace in dark blue and the TAMRA-based trace in light blue. The data demonstrate that the reaction pathway of the two pre-mRNA fragments can be independently investigated in one RNA-substrate molecule. Other multiple fluorophore-labeling strategies are also possible. The two CE/LIF-traces further show that both fluorescence signals converge for the product peak and all ligated intermediates confirming the re-ligation of the 5′- and 3′-pre-mRNA Cl-fragments after the catalytic conversion. (**B**) ‘One pot’ U-insertion and U-deletion RNA-editing. The cartoon depicts a trimeric 5′-TAMRA-substituted U-insertion RNA-substrate in red and a trimeric 5′-FAM-modified U-deletion RNA-substrate in blue. Both hybrid-RNAs were edited in the same reaction vial followed by automated CE/LIF-detection. Bar-plots show the percental abundance of all reaction intermediates and products in relation to the amount of input-RNA (from n = 3 experiments). The ability to monitor both editing reactions in one sample further reduces the sample size in HTS-screening experiments. FI = fluorescence intensity, RFU = relative fluorescence unit, EM = electrophoretic migration time in sec.

The structure-conservative characteristics of the fluorophore-substituted oligoribonucleotides encouraged us to investigate whether the assay system can be improved by multiplexing, i.e. by using multiple fluorophore modifications. Since modern CE/LIF-instruments can monitor up to five different fluorophores in one electrophoresis run we specifically addressed two scenarios: (i) a dual fluorophore-labeling approach of the two pre-mRNA-mimicking oligoribonucleotides and (ii) a side-by-side analysis of both, U-insertion and U-deletion editing in one reaction vial. Representative examples of the generated data-sets are shown in Figure 5. By using a 5′-FAM-substituted U-deletion 5′-Cl fragment in combination with a 3′-TAMRA-modified U-deletion 3′-Cl oligoribonucleotide the conversion of both pre-mRNA fragments into the same partially and fully-edited reaction products is unequivocally demonstrated (Figure 5A). Both, the FAM- and TAMRA-signals overlap for all partially and fully edited U-deletion products, while the two educts and all intermediates are well separated from each other. At the same time, the experiment scrutinizes the reaction pathway of both pre-mRNA fragments and corroborates that only the 5′-pre-mRNA fragment acts as source for the –1U, –2U, –3U and –4U intermediates. Similarly, by using a 5′-FAM-labeled U-deletion and a 5′-TAMRA-substituted U-insertion pre-mRNA/gRNA hybrid we demonstrate that both editing reactions can be performed in 'one-pot' (Figure 5B). The two CE-tracings are not influenced by each other and no qualitative or quantitative difference in comparison to the separate CE-runs is observed.

As a follow up we tested whether the two *in vitro* reactions can be downscaled to tailor the assay for high-throughput screening applications. This was done in a stepwise fashion using the signal to noise ratio (S/N) of the CE-electropherograms as a proxi. As lower limit we determined 2.5–10 fmol of each, editosomes and fluorophore-modified pre-mRNA/gRNA-hybrid in a reaction volume of 4μL. At these conditions signal intensities remained ∼300-fold over background for high intensity peaks and 50-fold over background for low intensity peaks. Because of the minute amounts of protein and RNA in the samples, reactions were stopped by directly adding formamide thereby eliminating the time-consuming phenol and EtOH-treatment steps. Furthermore, the small assay volume enables the usage of multiwell plates, which makes the test suitable for HTS-experiments. As a statistical HTS-quality score (39) we determined a mean screening window coefficient (Z’) of 0.6 confirming the aptness of the assay for HTS-screening purposes. Using the downscaled assay conditions, it is possible to quantitatively monitor 5000 chemical compounds on their influence on both RNA-editing reactions with only 10 μg (12.5 pmol) of editosomes and 2 μg (2 × 50 pmol) of fluorophore-modified pre-mRNA/gRNA–hybrid RNAs in just 78 h, using a 96-capillary CE-instrument.

Lastly, we scrutinized the kinetics of the catalytic conversion. Supplementary Figure S4 shows as an example the CE/LIF-traces of a U-deletion editing reaction, incubated for 5 min up to 180 min. Reaction intermediates and products can be identified as early as 5 min. At 50 min, the processed/input RNA-ratio approaches a value of 50% demonstrating that the reaction time can be shortened much below the standard 3 h of incubation.

### Optimization II—RNA-substrate chemistry

**Figure 6.**
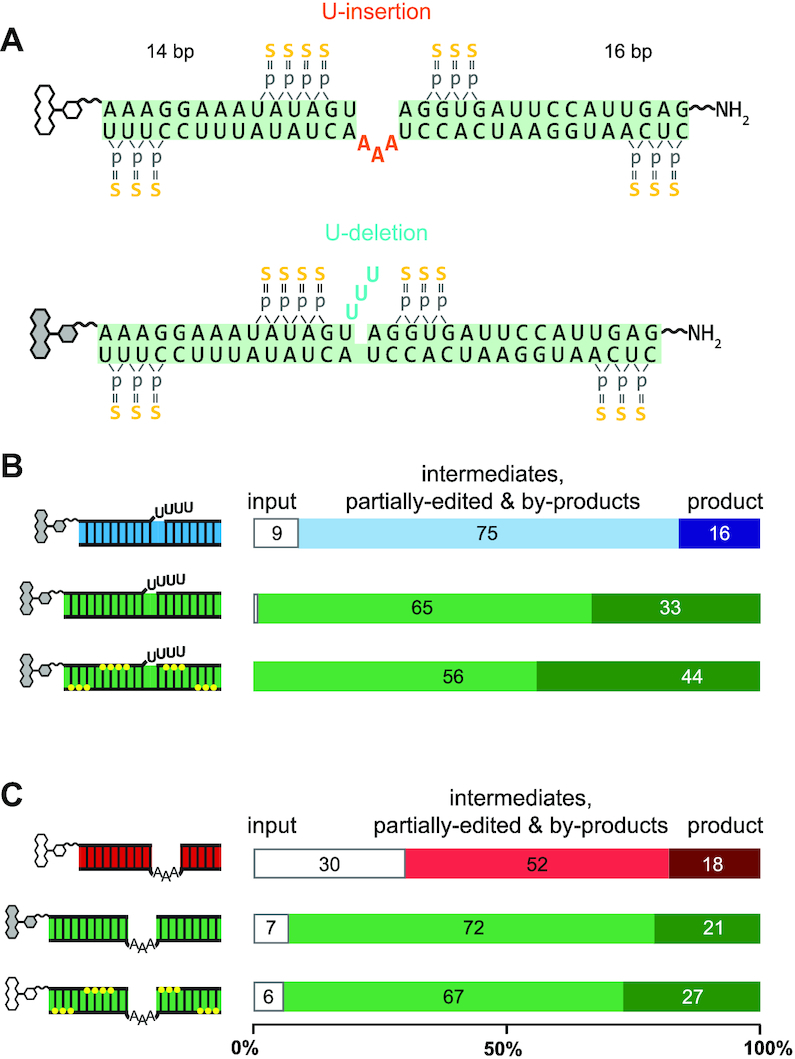
Pre-mRNA/gRNA hybrid-RNA optimization. (**A**) Sequence representation of the non-natural pre-mRNA/gRNA hybrid RNAs. Top: U-insertion hybrid-RNA. Bottom: U-deletion hybrid-RNA. Upper sequence strands represent the 5′ and 3′ pre-mRNA cleavage fragments and lower strands are gRNA molecules. The 14bp and 16bp-long helical RNA-elements flanking the editing sites are identical in both trimeric RNAs. Guide RNA nucleotides dictating the insertion of 3 U's are in red. The 3 U's that are deleted are in blue. Phosphorothioate (PS) modifications are in yellow and fluorophore positions are shown as chemical ring systems (white = TAMRA, gray = FAM). (**B**) Cartoons of U-deletion-type pre-mRNA/gRNA hybrid RNAs. Top: standard hybrid-RNA (blue); center: non-natural hybrid-RNA (green); bottom: non-natural, PS-modified RNA (green + yellow dots indicating PS-positions). Bar-plots to the right summarize the FIDE-assay results (from *n* = 5 experiments) specifying the percental amounts of U-deletion product, of intermediates, partially edited RNAs and by-products and of unprocessed input-RNA. (**C**) Cartoons of U-insertion-type pre-mRNA/gRNA hybrid-RNAs. Top: standard hybrid-RNA (red); center: non-natural RNA-hybrid (green); bottom: non-natural, PS-modified RNA (green + yellow dots indicating PS-positions). Bar-plots to the right summarize the FIDE-assay results (from *n* = 5 experiments) specifying the percental amounts of U-insertion product, of intermediates, partially edited RNAs and by-products and of unprocessed input-RNA. For both, the U-deletion and the U-insertion reaction, the non-natural, PS-modified RNAs represent the most efficient substrate molecules.

To further improve the FIDE-assay we focused on two additional aspects: (i) the ribonucleolytic stability of the substrate RNAs and (ii) possible sequence context effects because the U-deletion and U-insertion substrate RNAs are of different sequence. Since editosomes are purified from whole cell lysates co-purifying and/or contaminating RNases can be challenging (e.g. see Supplementary Figure S5A). The problem is even more pronounced if only partially purified mitochondrial (mt)-extracts are used as editosome source, despite the fact that mt-extracts are better suited for large-scale screening purposes due to time and budgetary concerns. To reduce the RNase sensitivity and, at the same time, to avoid any RNA-sequence bias we first modified the sequence of the pre-mRNA/gRNA hybrid to a non-natural sequence not found in the *T. brucei* mitochondrial RNA genome. In a second step we introduced multiple, RNase-resistant phosphorothioate bonds. The trimeric RNA is of identical sequence for both, the U-insertion and U-deletion reaction and differs only at the editing site where either three U-nt are inserted or deleted (Figure 6A). Phosphorothioates (PS) substitute a sulfur atom for a non-bridging oxygen in the phosphate backbone and this renders the internucleotide linkage nuclease resistant. Altogether 13 phosphorothioates were co-synthetically introduced into the trimolecular hybrid-RNAs: 4, respectively 3 PS were positioned on either side of the pre-mRNA editing domains and six additional phosphorothioates were placed next to the two termini of the gRNA-oligonucleotides (Figure 6A). Incubation of the PS-derivatized oligoribonucleotides with editosomes in the absence of gRNAs verified their RNase-resistant characteristics (Supplementary Figure S5B/C). While up to 70% of the unmodified 5′-pre-mRNA oligonucleotides were degraded within 3 h at 27°C, 70–80% of the PS-modified RNAs remain stable over the same period. Furthermore, we verified that the synthetic, PS-modified oligoribonucleotides are able to form trimeric pre-mRNA/gRNA complexes. The different RNA-oligonucleotides anneal with reduced efficiencies (between 65% and 87%) (Supplementary Figure S6), however, the trimeric RNAs exhibit thermodynamic stabilities very similar to their non-modified counterparts (Supplementary Figure S7 and Supplementary Table S1). Finally, we tested the PS-substituted, non-natural pre-mRNA/gRNA hybrids as editing substrates. Figure 6B/C demonstrates that both RNA-hybrids are properly processed by editosomes generating all expected partially- and fully edited products as well as all intermediates and by-products of the reaction. A reference chart of all expected RNA-species is given in Supplementary Figure S2. A comparison of the product/input (p/i)-ratio identifies that the non-natural pre-mRNA/gRNA-hybrids outperform the trypanosome-based pre-mRNA/gRNA sequences between 10- and 20-fold, which together with their improved RNase-stability makes them well suited as substrates in HTS-experiments.

### Validation—screening for inhibitory UTP-analogs

**Figure 7.**
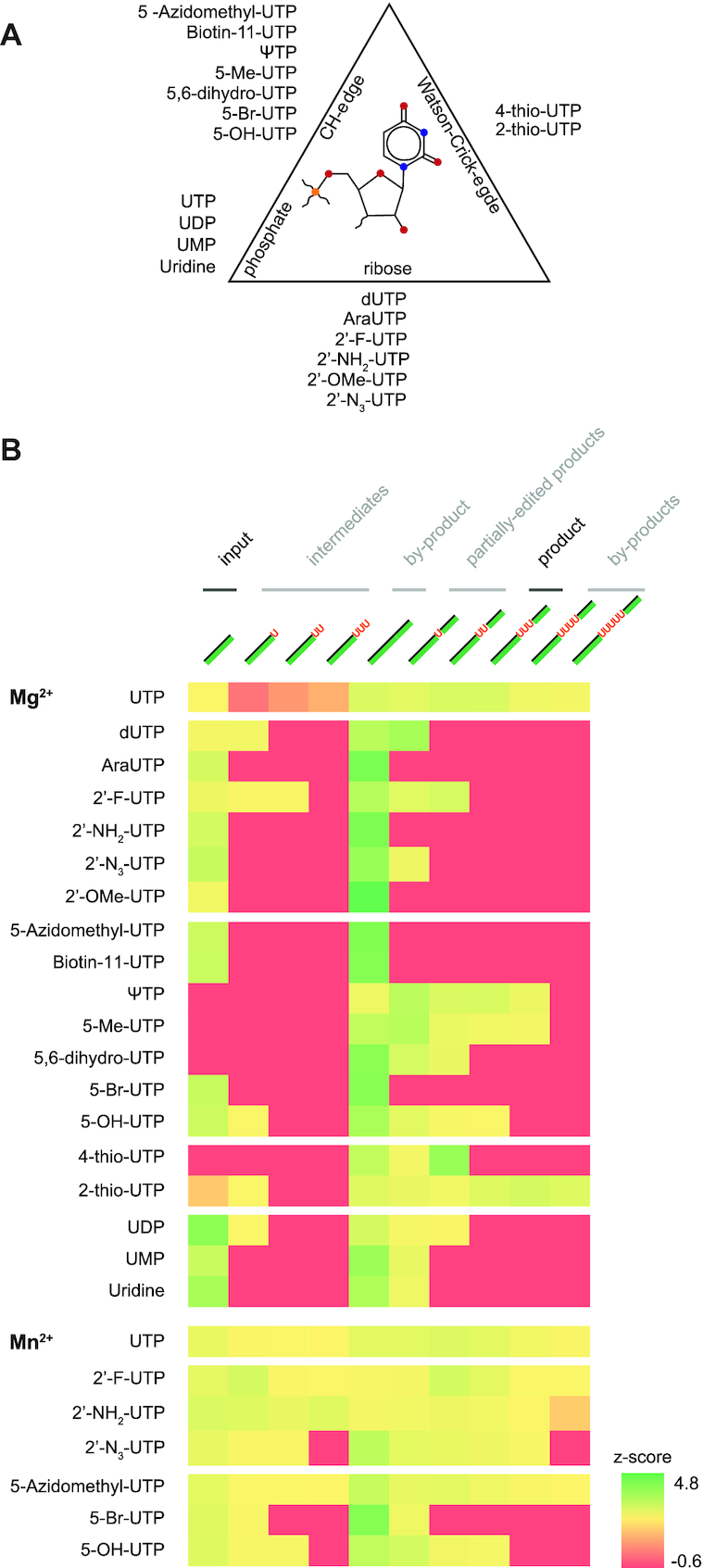
Inhibition of U-insertion RNA-editing by UTP-analogs. (**A**) Tested UTP-analogs are listed next to a ball-and-stick representation of UMP to positionally categorize the different compounds as CH-edge-, Watson/Crick edge-, phosphate- or ribose-interfering. (**B**) *Z*-score representation of the FIDE-assay results for the reaction input, all reaction intermediates and partially edited products as well as the fully edited product and reaction by-products (*Z* = *x* – μ/∂ with *x* = score, μ = mean, ∂ = standard deviation). A reference chart of the different RNA-species is given in Supplementary Figure S2. The heat-map block at the bottom summarizes the incorporation of UTP-analogs in the presence of Mn^2+^ instead of Mg^2+^.

To demonstrate the high-throughput potential of the FIDE-assay, we performed a small-scale, proof-of-principle screening experiment. For that we made use of the non-natural U-insertion pre-mRNA/gRNA-substrate described above and examined whether U-nucleotide analogs might interfere with the U-insertion reaction. Both, pyrimidine ring-modified compounds (5-CH_3_-UTP, 5-Br-UTP, 5-OH-UTP, 4-S-UTP, 2-S-UTP, 5-biotin-UTP, 5-CH_3_N_3_-UTP etc.) as well as ribose-modified analogs such as 2′-CH_3_O-UTP, 2′-NH_2_-UTP, 2′-F-UTP, 2′-N_3_-UTP and Ara-UTP were tested. A list of all compounds is shown in Figure 7A. In addition, we probed the influence of Mg^2+^- and Mn^2+^-cations in the reaction. Altogether 26 samples were tested, which on a 48 multicapillary CE/LIF-instrument can be assayed in just 1.5h generating 260 quantitative data points on the formation of the fully edited product and on every reaction intermediate. As shown in Figure 7B the data confirm that UTP is the preferred substrate of the reaction. Furthermore, the data reveal that the ribose 2′-OH group is more important in the catalytic conversion than the majority of substituents at the CH- and Watson/Crick-edges of the U-nucleobase. All 2′-ribose modifications inhibit the formation of the fully edited (+3U) product and only 2′-F-UTP is to some degree tolerated (forming a +2U product). By contrast, multiple responses were detected for all base-modifications. They range from complete inhibition by 5-Br-UTP to different degrees of misediting by 5-CH_3_-UTP (+4Us) and 2-S-UTP (+4 and +5U′s). Mn^2+^-ions have an enhancing effect, which can result in the incorporation of otherwise non-inserted UTP-analogs. Together, the data demonstrate the versatility of the FIDE-*in vitro* system by enabling a comprehensive and quantitative inhibitor screening in a very short time.

## CONCLUSION

In summary, we have converted the standard *in vitro* assay to monitor editosome function in African trypanosomes and related organisms into a high-throughput analysis format. Core attribute of the revised assay is the usage of fluorophore-labeled RNA-editing substrate RNAs. This enables the automated electrophoretic separation of the products of the catalytic conversion using state-of-the-art, high-throughput capillary electrophoresis instruments coupled with laser-induced fluorescence readout systems. As such the assay generates quantitative data of all educts, intermediates and products and by using multicapillary instruments it can be performed in a highly parallel format. We optimized the assay by downscaling the required material quantities as well as the reaction volume to adapt the procedure to all available multiwell-plate formats. Additional improvements include the usage of non-natural, RNase-resistant RNA-substrates, which enable large-scale screening experiments using crude mitochondrial extracts instead of highly purified editosome preparations. We also verified that the experimental setup tolerates multiplex-type, fluorophore-labeling strategies, which expands the parallel analysis-capacity of the assay further. As demonstrated in a pilot screening experiment, the assay is applicable to larger sample-size studies to probe specific mechanistic aspects of the RNA-editing reaction. However, the assay is especially well suited to conduct large-scale, high-throughput screening experiments to identify small molecule inhibitors. Since RNA-editing is an essential biochemical pathway in African trypanosomes, an assay format that permits a robust, quantitative as well as time- and material-resourceful analysis should be helpful in the search for novel therapeutics to challenge African trypanosomiasis and related diseases.

## Supplementary Material

gkaa658_Supplemental_File
