# Supplementary material for: Analyzing editosome function in high-throughput
Source: Nucleic Acids Res. 2020 Aug 5;48(17):e99. doi: 10.1093/nar/gkaa658 (PMC7515698; doi:10.1093/nar/gkaa658)
Supplement: gkaa658_Supplemental_File [file gkaa658_supplemental_file.pdf]

Supplementary Material

Analyzing editosome function in high-throughput

Cristian Del Campo, Wolf-Matthias Leeder, Paul Reißig, H. Ulrich Göringer
Molecular Genetics, Technical University Darmstadt, Schnittspahnstr. 10, 64287 Darmstadt, Germany

Supplementary Figures:

Figure S1.	Oligoribonucleotide quality assessment
Figure S2	Assignment and categorization of all FIDE-derived RNA-species
Figure S3.	Comparison of the fluorophore CE/LIF-based RNA-editing assay with the standard isotope labeling-based assay
Figure S4.	Kinetic of the catalytic conversion
Figure S5.	RNA-substrate susceptibility to RNases
Figure S6.	Annealing of non-natural pre-mRNA/gRNA-hybrid RNAs
Figure S7.	Thermodynamic stabilities of non-natural pre-mRNA/gRNA-hybrid RNAs

Supplementary Tables:

Table S1.	Summary of half-maximal melting transitions (T_m-values) for the different pre-mRNA/gRNA-hybrid RNAs

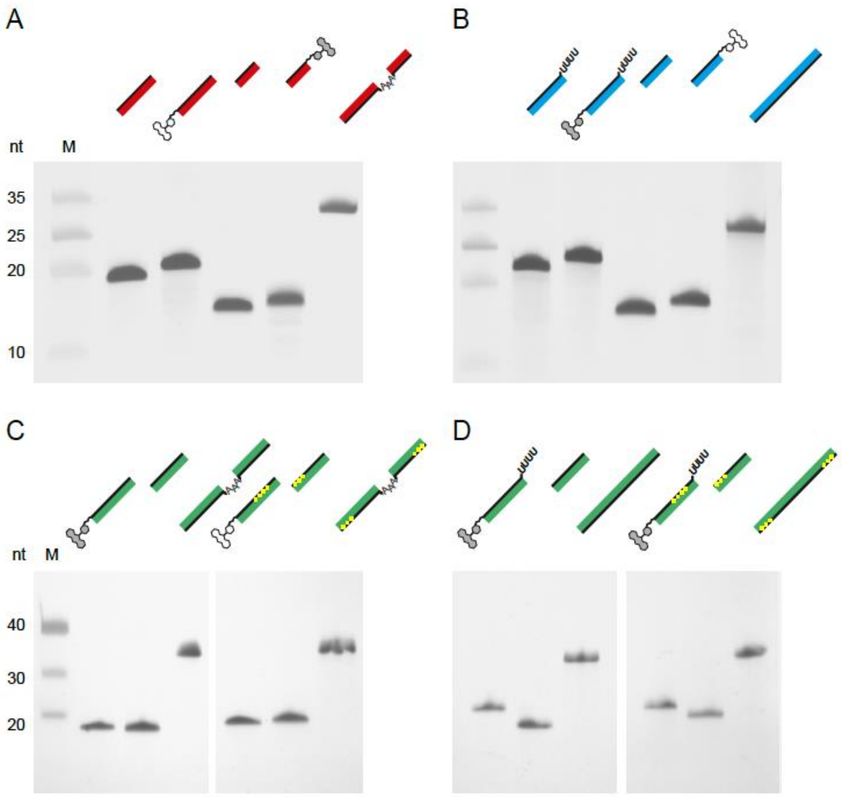

Figure S1. Oligoribonucleotide quality assessment. Chemically synthesized oligoribonucleotides were postsynthetically scrutinized by gel-electrophoresis in 8M urea-containing, 15% (w/v) polyacrylamide gels followed by Toluidine Blue O staining. From left to right: (A) 5'-Cl18, 5'-TAMRA_Cl18, 3'-Cl13, 3'-Cl13_FAM, gRNA$_{ins}$. (B) 5'-Cl22, 5'-FAM_Cl22, 3'-Cl15, 3'-Cl15_TAMRA, gRNA$_{del}$. (C) 5'-FAM_synCl14, 3'-synCl16, syngRNA$_{ins}$, 5'-FAM_modCl14, 3'-modCl16, modgRNA$_{ins}$. (D) 5'-FAM_synCl17, 3'-synCl16, syngRNA$_{del}$, 3'-modCl16, modgRNA$_{del}$, 5'-FAM_modCl17. RNA-sequences are listed in the Materials and Method section. Fluorophore modifications are shown as chemical ring systems (white=TAMRA, grey=FAM). M=marker. Yellow dots=phosphorothioate-modifications. Stained gels were densitometrically analyzed. All RNA-preparations are ≥97% pure.

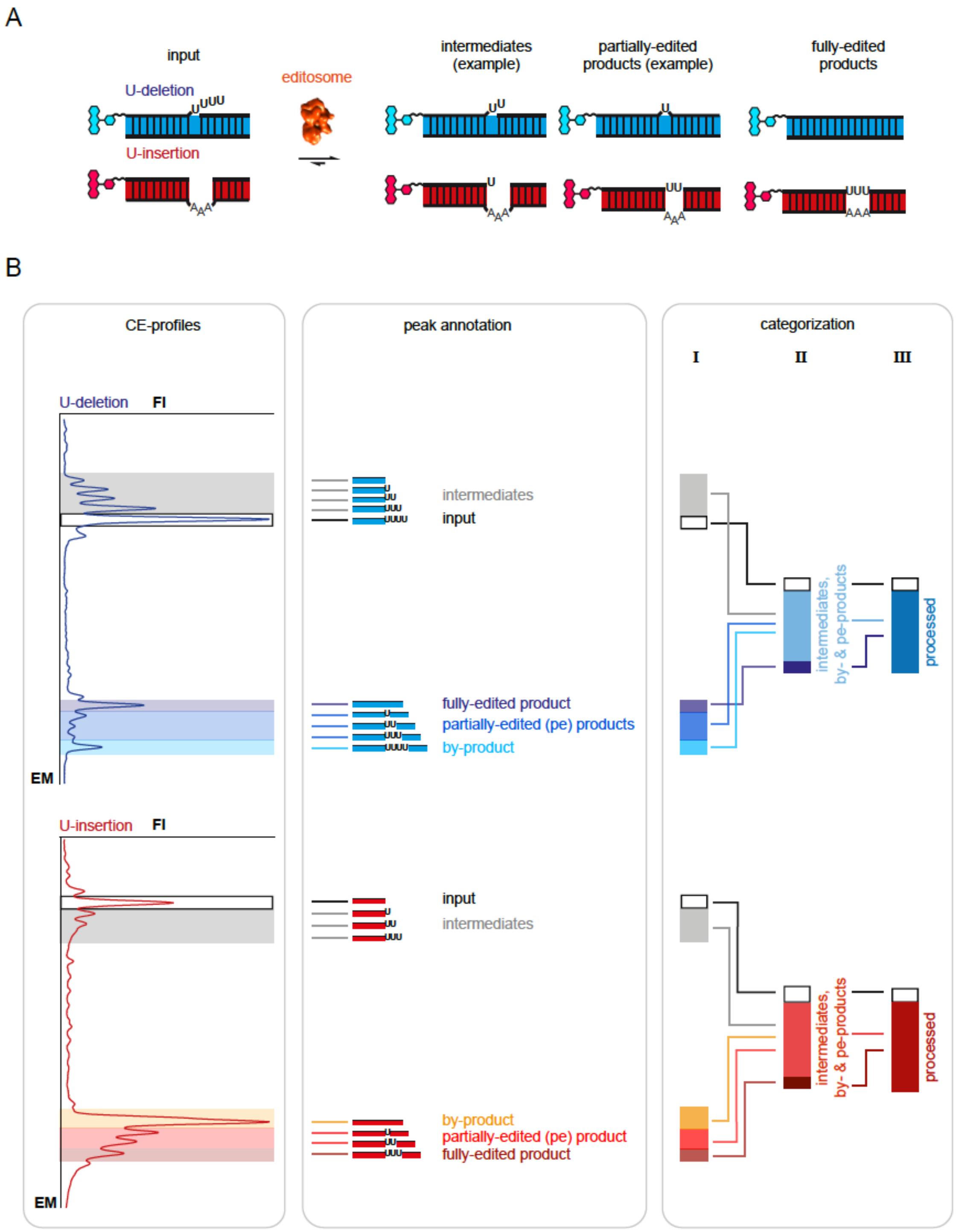

Figure S2. Assignment and categorization of all FIDE-derived RNA-species. (A) Cartoons of a U-deletion (blue) and U-insertion (red) FIDE-assay depicting the two trimolecular pre-mRNA/gRNA-input RNAs along with examples of reaction intermediates, partially-edited RNAs and the fully edited RNA products. (B) Left: Representative CE-profiles of FIDE U-deletion (blue) and U-insertion (red) assays (FI= fluorescence intensity; EM= electrophoretic migration time). Center: peak assignment of all RNA-species using the categories: input RNA, fully-edited product-RNA, reaction intermediate, partially edited (pe)-RNA and by-product. Right: Grouping scheme of the different RNA-species using three levels of granularity from fine (I) to medium (II) to coarse (III).

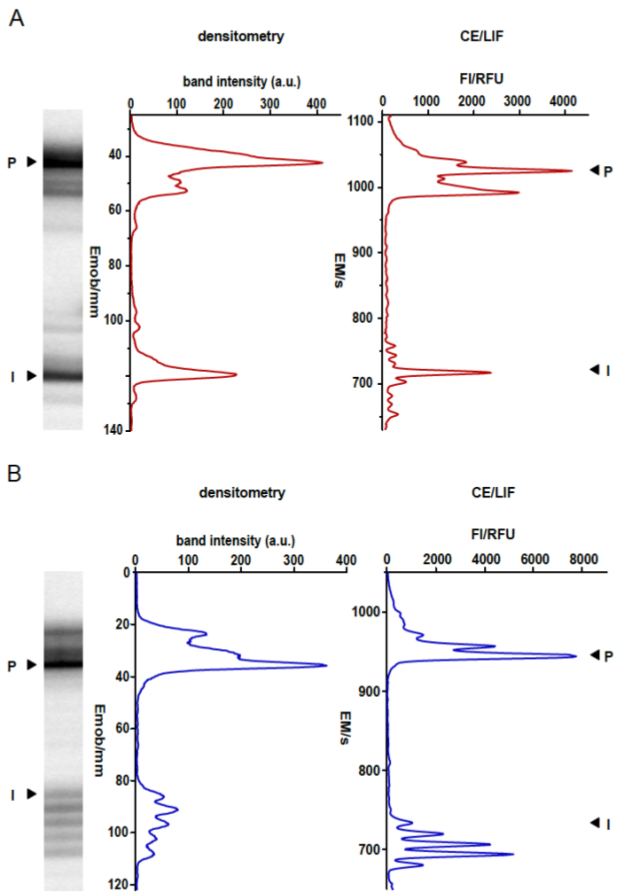

Figure S3. Comparison of the fluorophore CE/LIF-based RNA-editing assay with the standard isotope labeling-based assay. (A) U-insertion RNA editing. (B) U-deletion RNA editing. (^{32}P)-labeled RNA-reaction products were electrophoretically separated in 8M urea-containing 18% (w/v) polyacrylamide gels and were densitometrically quantified. Fluorophore-labeled substrate RNAs were analyzed by CE/LIF. (I)=input RNA. (P)=fully edited product-RNA. The experiments compare with Pearson correlation coefficients of p=0.87 for the U-insertion assays and with p=0.86 for the U-deletion assays. FI=fluorescence intensity, RFU=relative fluorescence unit, EM=electrophoretic migration time in seconds and Emob=electrophoretic mobility in mm.

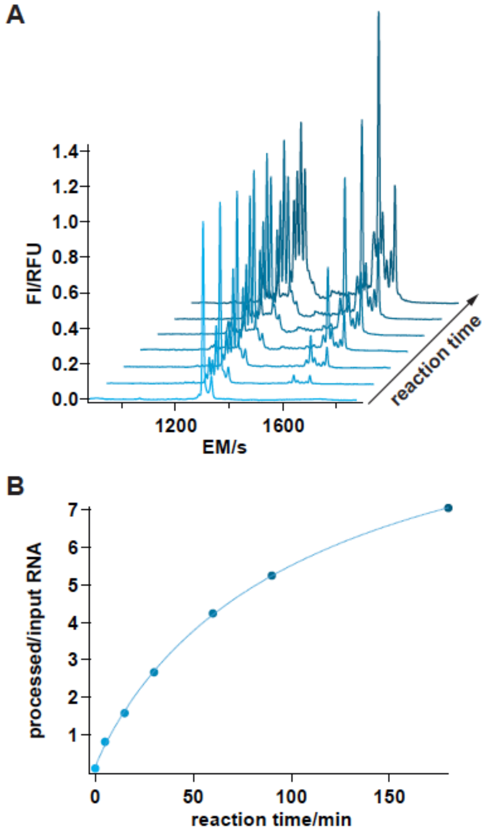

Figure S4. Kinetic of the catalytic conversion. Time course of a U-deletion FIDE-assay for reaction times of 0, 5, 15, 30, 60, 90 and 180min. (A) CE/LIF-traces of the different samples from 0min (front) to 180min (back). EM=electrophoretic migration time in seconds**.** FI=fluorescence intensity. RFU=relative fluorescence unit. (B) Plot of the processed/input RNA-ratio over time. Data points are curve-fitted to the Hill-equation with a chi-square of 0.015.

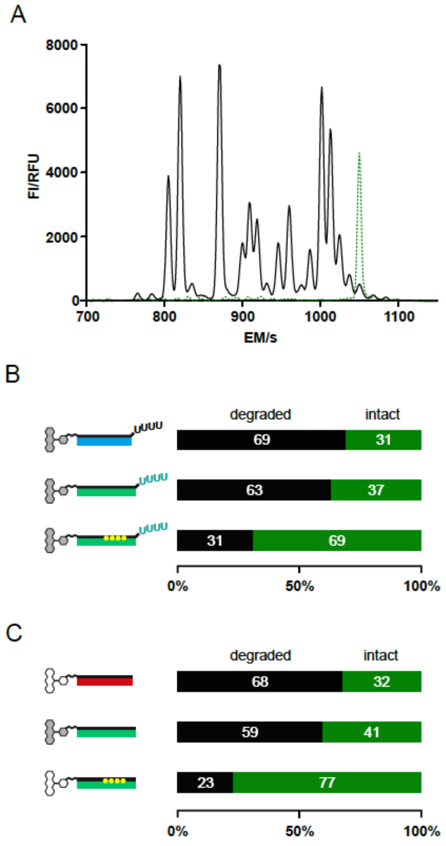

Figure S5. RNA-substrate susceptibility to RNases. Ribonucleolytic degradation of fluorophore-modified 5'-pre-mRNA cleavage fragments (Cl) upon incubation with editosomes. (A) CE-trace of a fully degraded 5'-Cl fragment (black). Dashed trace in green: input 5'-Cl-RNA). (B) U-deletion 5' Cl-fragments. Top: standard 5'-Cl RNA-oligonucleotide (blue). Centre: non-natural 5'-Cl RNA green. Bottom: non-natural, phosphorothioate (PS)-modified 5'-Cl RNA (green + yellow dots marking PS-positions). (C) U-insertion 5' Cl-fragments. Top: standard 5'-Cl RNA-oligonucleotide (red). Centre: non-natural 5'-Cl RNA (green). Bottom: non-natural, phosphorothioate (PS)-modified 5'-Cl RNA (green + yellow dots marking PS-positions). Fluorophore positions are shown as chemical ring systems (white=TAMRA, grey=FAM). Bar-plots display the percentage of intact (green) and degraded RNA (black) after a 3h incubation at 27°C (from n=3 experiments).

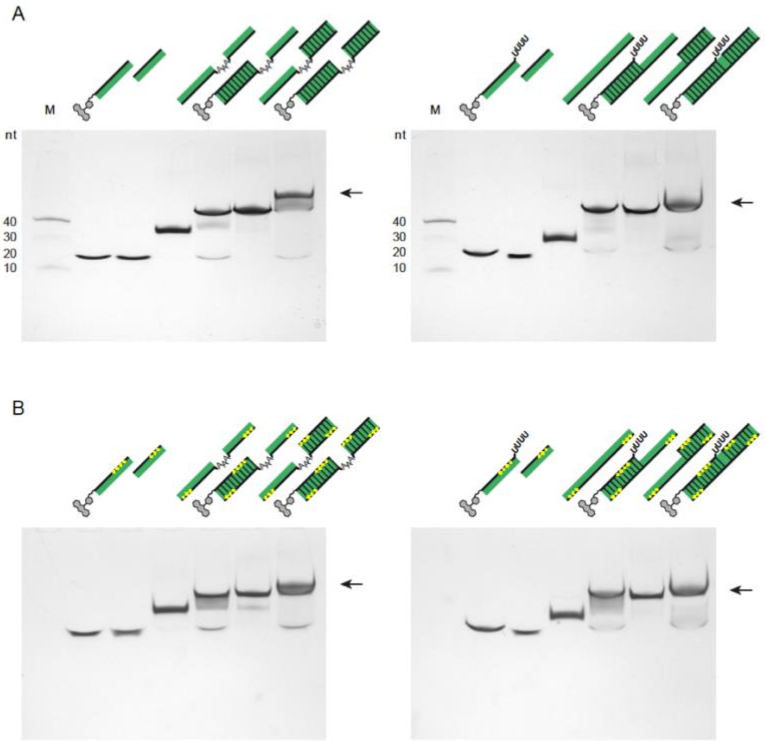

Figure S6. Annealing of non-natural pre-mRNA/gRNA-hybrid RNAs. Gel-electrophoretic analysis of the formation of trimolecular pre-mRNA/gRNA hybrid RNAs for the non-natural pre-mRNA/gRNA editing substrates (A) and the non-natural, phosphorothioate (PS)-modified editing substrates (B). Left: U-insertion RNAs. Right: U-deletion RNAs. Fluorophore subtituents are shown as chemical ring systems in grey. Yellow dots mark PS-positions. All gel-electrophoretic separations show (from left to right) the 5'-pre-mRNA cleavage fragment (5'-Cl), the 3'-pre-mRNA cleavage fragment (3'-Cl) and the corresponding gRNA-oligoribonucleotide next to the two bimolecular complexes (5'-Cl/gRNA; 3'-Cl/gRNA) and the final trimolecular (5'-Cl/3'-Cl/gRNA) annealing product (arrow). Annealing efficiencies vary between 65% and 87%.

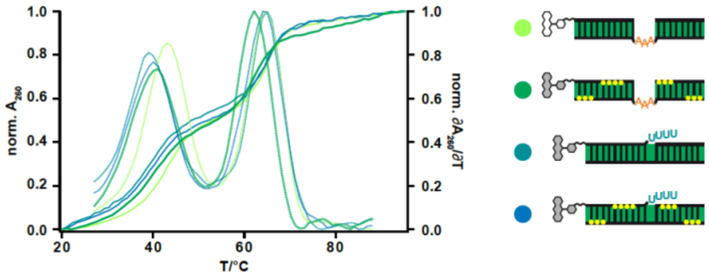

Figure S7. Thermodynamic stabilities of non-natural pre-mRNA/gRNA-hybrid RNAs. Comparison of the UV-melting (A_{260}=f(T)) and 1_{st}-derivative profiles ($\delta A_{260}/\delta T$=f(T)) of non-natural pre-mRNA/gRNA hybrid-RNAs and non-natural, phosphorothioate (PS)-modified pre-mRNA/gRNA hybrid-RNAs. Light/dark green: U-insertion RNAs. Light/dark blue: U-deletion RNAs. Fluorophore positions are shown as chemical ring systems (white=TAMRA, grey=FAM). Yellow dots indicate PS-modifications. Halfmaximal melting temperatures (T_m) are listed in Table S1.

Table S1. Summary of half-maximal melting transitions (T_m-values) for the different pre-mRNA/gRNA-hybrid RNAs. Red: U-insertion RNAs. Blue: U-deletion RNAs. Green: non-natural U-insertion and U-deletion RNAs. T_m-values are in degree Celsius and are derived from ≥2 UV-melting measurement with SD-values ≤2%. Fluorophore positions are shown as chemical ring systems (white=TAMRA, grey=FAM). Yellow dots indicate phosphorothioate (PS)-modifications

RNA-substrate designation		T_m (°C) 1st trans.	T_m (°C) 2nd trans.
Standard U-insertion	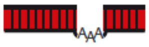	47.0	68.0
	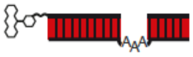	47.0	68.0
	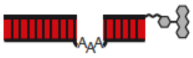	46.5	67.3
	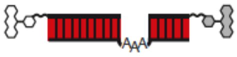	47.0	68.0
Standard U-deletion	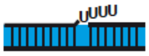	55.0	64.0
	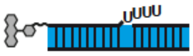	55.0	63.7
	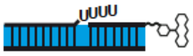	55.3	63.6
	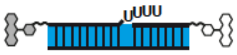	56.0	64.5
Non-natural U-insertion	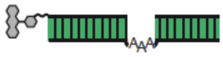	43.0	65.0
Non-natural, PS-modified U-insertion	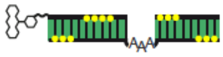	41.0	62.0
Non-natural U-deletion	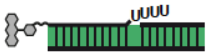	39.0	65.0
Non-natural, PS-modified U-deletion	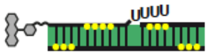	41.1	64.1
